# Intracranial meningiomas at a tertiary hospital: Spectrum of MRI findings with histopathologic correlation

**DOI:** 10.4102/sajr.v28i1.2812

**Published:** 2024-03-27

**Authors:** Jacobus A. Pienaar, Jacob Varghese

**Affiliations:** 1 Department of Diagnostic Radiology, Faculty of Health Sciences, University of the Witwatersrand, Johannesburg, South Africa; 2 Department of Diagnostic Radiology, Klerksdorp/Tshepong Hospital Complex, Klerksdorp, South Africa

**Keywords:** neuroradiology, meningioma, intracranial neoplasm, magnetic resonance imaging, histology, neurosurgery

## Abstract

**Background:**

Intracranial meningiomas consist of a heterogenous group of histological subtypes, some of which are rare. Data that may play an important role in neurosurgical decision-making regarding the incidence and MRI features of these histological subtypes in the South African population groups, are lacking.

**Objectives:**

This study aimed to assess the spectrum of MRI findings and histological subtypes of meningiomas in the South African context, with the goal of improving the paucity of literature on the topic.

**Method:**

A retrospective review of the MRI features of 41 cases of histologically confirmed intracranial meningiomas was performed at a tertiary hospital level. Imaging features were audited and correlated with histological subtypes during statistical analysis.

**Results:**

Eleven different histological subtypes of meningioma were encountered. World Health Organization (WHO) Grade I meningothelial meningiomas were the most common histological subgroup. Overall, meningiomas were found to be predominantly isointense to grey matter on T1-weighted imaging, irrespective of the histological subtype, with greater signal variability on T2-weighted imaging. Morphologies of specific subtypes are in keeping with the literature.

**Conclusion:**

Analysis of this series of intracranial meningiomas did not demonstrate statistically significant differences in MRI features between histological subtypes to allow for accurate preoperative prediction of meningioma subtype or WHO grade. This highlights the importance of definitive histopathological diagnosis rather than over-reliance on presumed benign imaging features.

**Contribution:**

This original research article discusses the impact of histological subtype on the MRI appearance of intracranial meningiomas, with the aim to improve the paucity of literature on the subject in the context of the South African population.

## Introduction

Intracranial meningiomas are common lesions encountered during neuroimaging.^1,2,3^ These tumours consist of a heterogenous group of histological subtypes, some very rare, that many radiologists and neurosurgeons may be unfamiliar with, and an incorrect diagnosis based on atypical imaging appearances may impact neurosurgical planning.^2^ The accuracy of MRI for the identification of intracranial meningiomas is estimated at close to 95%, making it an ideal tool to investigate these tumours.^4^

Data in the African, and more specifically, South African populations, regarding the incidence and MRI features of meningiomas, and especially of the more uncommon histological subtypes, is still somewhat lacking. Although many of the histological subtypes can show overlap and even identical MRI features, international studies have shown that some may have certain characteristics on specific MRI sequences that could potentially allow for a more accurate degree of preoperative identification, which may in turn aid with more efficient neurosurgical planning.

The aim of this study is to document the spectrum of MRI findings and corresponding histological subtypes of meningiomas in a local population, with the goal of improving the paucity of literature on the topic in the South African context.

## Materials and methods

A retrospective, cross-sectional, single-centre audit with descriptive and correlative components was performed at a tertiary hospital level. Adult patients with histologically confirmed intracranial meningiomas, who had undergone brain MRI at the centre over a 4-year study period from 01 July 2018 to 01 July 2022, formed the study population. Of the 96 initially identified cases with presumed intracranial meningiomas based on MRI, 41 cases met the inclusion criteria of histological confirmation; the remaining 55 cases did not have documented operative intervention with sampling for histological evaluation.

All MRI studies were performed on a 1.5 Tesla MRI scanner (Toshiba Vantage Titan, Japan) with a basic neuroimaging protocol, which included, but was not limited to, axial T1-weighted imaging (T1-WI), T2-weighted imaging (T2-WI), T2-fluid attenuation inversion recovery (FLAIR) and T1-WI postcontrast sequences (obtained only for patients who had a normal renal function, defined as an estimated glomerular filtration rate [eGFR] greater than 60). The contrast medium used was a gadolinium-based agent (gadopentetate dimeglumine, brand name Magnevist, Bayer), intravenously administered via a peripheral line at the standard adult bolus dose of 10 mL. In addition, diffusion-weighted imaging (DWI), apparent diffusion coefficient (ADC) and gradient echo (GRE) sequences were also variably performed and analysed where available.

Reading of the axial brain MRI was standardised using an MRI reader sheet with predefined descriptive terminology to report the imaging findings, which reflected a thorough assessment of identified MRI lesion characteristics. Relevant data collected comprised a morphological assessment of each lesion, an assessment of its signal characteristics on various standard MRI sequences, the pattern of postcontrast lesion enhancement, and other features associated with the lesion. Morphological assessment of the meningioma included an evaluation of the macroscopic shape (described as flat or ‘en plaque’ vs. round or ‘globose’), and definition of the margins of the lesions (well circumscribed or poorly circumscribed), composition (solid, homogenous appearance or heterogeneity because of variable soft tissue signal intensity, cystic or necrotic areas) and the pattern of internal vascularity (as ‘sunburst or spoke wheel’, disorganised or random and minimal or absent). Signal characteristics in relation to the cerebral parenchymal grey matter were assessed on T1-WI, T2-WI and FLAIR sequences (reported as isointense, hypointense or hyperintense compared to cerebral cortical grey matter). Where available, the presence of blooming artefact on GRE sequences and the presence of diffusion restriction assessed on DWI-ADC sequences were also documented. The postcontrast appearance (as non-enhancing, mildly enhancing or avidly enhancing) was assessed. Other related findings documented included the presence of perilesional brain oedema (subjectively graded as absent, mild, moderate or severe), the presence of associated focal osseous changes (as absent, osseous erosion, invasion, destruction or hyperostosis) and the presence of a ‘dural tail sign’ (as present or absent). Locations of the meningiomas were documented as cerebral convexity, parafalcine, sphenoid wing, olfactory groove, planum sphenoidale, sellar, cerebellopontine angle and craniocervical junction.

Relevant additional demographic and histological data collected included the patient’s age, gender, histological diagnosis, specified histological subtype if provided and World Health Organization (WHO) grading of the meningioma.

### Statistical analysis

Statistical analysis was performed using the IBM^®^ SPSS^®^ version 28. Descriptive statistics in terms of frequencies and percentages were calculated for the categorical data, and means with standard deviation were calculated for numerical data which were normally distributed. The Chi-Square and Fisher’s exact tests were used to compare frequencies. Statistical significance was determined by a *p*-value of < 0.05. For the analysis of overall demographic and signal characteristics, as well as morphological and perilesional features of lesions, all cases were included in calculating percentages (total *n* = 41). For comparative assessment between specific histological subtypes, percentages and *p*-values were calculated using only cases with specified histological subtypes (total *n* = 37). Additional totals used are explicitly stated.

### Ethical considerations

Ethical clearance to conduct the study was obtained from the centre’s Chief Executive Officer (CEO) and provided by the University of the Witwatersrand’s Committee of Human Research of the Faculty of Health Sciences (ethics clearance no. M220729) prior to the initiation of the study. As a result of the retrospective nature of the study, no direct patient contact or informed consent from the patients themselves was required. Patient anonymity, data safety and confidentiality were ensured at all times.

## Results

In terms of demographics, the ages of the patients with intracranial meningiomas ranged from 31 years to 74 years, with 58.5% older than 50 years. The mean patient age at diagnosis was 53 years. The gender distribution was predominantly female (85.4%), with a female to male ratio of 5.8:1.

The WHO grading of the intracranial meningiomas showed overwhelmingly benign tumours (WHO Grade I) with a small minority of atypical (WHO Grade II) and malignant (WHO Grade III) cases. A variety of different histological subtypes were identified. World Health Organization Grade I meningiomas can be further divided into traditionally commonly encountered (73.1%, *n* = 30) and uncommonly encountered (9.6%, *n* = 4) groups (Table 1). Four of the total cases (*n* = 41) were pathologically graded as WHO Grade I meningiomas, but no definitive subtype was specified.

**TABLE 1:** Frequencies and percentages of the varying histological subtypes of intracranial meningiomas encountered, subdivided by WHO grade, as well as specifying common and uncommon benign subtypes.

WHO grading and histological subtype	Frequency	Percentage
**WHO grade I**	38	92.5
Common benign subtypes	30	73.1
Meningothelial	24	58.5
Transitional	5	12.2
Fibroblastic	1	2.4
Uncommon benign subtypes	4	9.6
Microcystic and angiomatoid	1	2.4
Microcystic	1	2.4
Psammomatous	1	2.4
Secretory	1	2.4
Unspecified benign subtypes	4	9.8
**WHO grade II**	2	4.8
Atypical	1	2.4
Chordoid	1	2.4
**WHO grade III**	1	2.4
Anaplastic	1	2.4
**Total**	**41**	**100**

**FIGURE 1:**
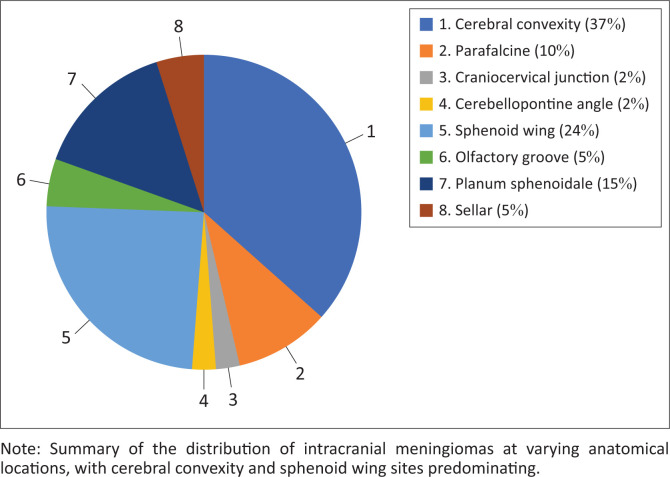
Anatomical locations of intracranial meningiomas (*n* = 41).

Meningiomas were found in a variety of intracranial locations. Cerebral convexity (37%) and sphenoid wing (24%) locations predominated (Figure 1). There were no statistically significant associations between different histological subtypes and specific intracranial locations (*p* = 0.82).

In terms of lesion morphology, the meningiomas were most commonly globose in shape (90.2%) with well circumscribed margins (85.4%). En plaque (9.8%) and poorly circumscribed (14.6%) lesions were seen in a minority of cases. All en plaque meningiomas were of the meningothelial subtype (*n* = 4), and these comprised 67% of the poorly circumscribed lesions, with poor marginal definition at their interface with the underlying bone. No other statistically significant associations were identified between histological subtype and the macroscopic form or marginal definition of the lesions (*p*-values of 0.98 and 0.55, respectively). Most meningiomas were homogenous and solid (61%) with some lesions showing heterogeneity because of variable internal soft tissue signal intensity (7.3%), internal calcification (14.6%) or cystic components (17.1%). No statistically significant associations were found between histological subtype and these aspects of lesional morphology, with WHO Grade I subtypes such as meningothelial meningiomas sometimes seen as heterogenous lesions because of cystic and calcific internal components. Most meningiomas did not show discernible internal flow voids (63.4%). A disorganised pattern of peripheral predominant vessels was seen in some cases (31.7%) with the classic sunburst, or spoke wheel pattern, encountered in very few cases (4.9%). No statistically significant association was identified between histological subtype and internal vascularity (*p* = 0.8). A summary of the data regarding these morphological characteristics is provided in Table 2.

**TABLE 2:** Morphological characteristics of the varying histological subtypes of intracranial meningiomas (total *n* = 41).

Histological subtype	Morphological characteristics of intracranial meningiomas								
	Macroscopic form		Margins		Composition		Vascularity		
	Globose	En plaque	Well circumscribed	Poorly circumscribed	Homogenous	Heterogenous	Minimal	Random	Sunburst
**WHO grade I**									
Meningothelial	20	4	19	5	12	12	17	6	1
Transitional	5	-	5	-	5	-	2	2	1
Fibroblastic	1	-	1	-	1	-	1	-	-
Secretory	1	-	1	-	1	-	1	-	-
Psammomatous	1	-	1	-	-	1	1	-	-
Microcystic and angiomatous	1	-	1	-	-	1	-	1	-
Microcystic	1	-	1	-	1	-	1	-	-
Unspecified	4	-	4	-	3	1	2	2	-
**WHO grade II**									
Chordoid	1	-	-	1	1	-	-	1	-
Atypical	1	-	1	-	1	-	1	-	-
**WHO grade III**									
Anaplastic	1	-	1	-	-	1	-	1	-

**Total**	**37**	**4**	**35**	**6**	**25**	**16**	**26**	**13**	**2**

Of the cases with T1-WI acquisitions (*n* = 40), most meningiomas across all histological subtypes were consistently T1-WI isointense (85%) compared to cortical grey matter. The T2-WI signal intensity of the meningiomas was much more variable, with both hyperintense (53.7%) and isointense (41.5%) appearances commonly encountered. All cases (*n* = 41) had T2-WI imaging preformed and showed T2-WI and FLAIR signal concordance. Frequencies of the differing T1-WI, T2-WI and FLAIR signal intensity, correlated with histological subtype, are displayed in Table 3. No statistically significant differences were identified between the T1-WI or T2-WI/FLAIR signal characteristics of common and uncommon histological subtypes (*p* = 0.12 and *p* = 0.28, respectively). Of the cases with available postcontrast T1 imaging (*n* = 39), the pattern of enhancement was overwhelmingly avid (94.9%), irrespective of the histological subtype. Of the 35 cases with DWI-ADC sequences preformed, diffusion restriction was encountered in 48.6% of cases; however, no statistically significant differences in terms of diffusion restriction between histologic subtypes were identified (*p* = 0.37). Unfortunately, GRE sequences were only preformed in a select number of cases (*n* = 29), too small in number to draw significant conclusions.

**TABLE 3:** Summary of the MRI signal characteristics of the varying histological subtypes of intracranial meningiomas on T1-WI, T2-WI, ADC/DWI and GRE sequences.

Histological subtype	T1-weighted Imaging			T2-weighted imaging			Diffusion restriction on ADC / DWI		Blooming on GRE	
	Hyperlntense	Hypointense	Isolntense	Hyperlntense	Hypointense	Isointense	Yes	No	Yes	No
**WHO grade I**										
Meningothelial	-	2	21	14	-	10	10	9	5	13
Transitional	-	2	3	2	1	2	1	4	2	-
Fibroblastic	-	-	1	1	-	-	1	-	-	-
Secretory	1	-	-	1	-	-	-	1	-	1
Psammomatous	-	-	1	-	1	-	-	1	1	-
Microcystic and angiomatous	-	1	-	1	-	-	1	-	-	-
Microcystic	-	-	1	1	-	-	-	1	-	1
Unspecified	-	-	4	-	-	4	2	1	-	1
**WHO grade II**										
Chordoid	-	-	1	1	-	-	-	1	-	-
Atypical	-	-	1	1	-	-	1	-	-	1
**WHO grade III**										
Anaplastic	-	-	**1**	-	-	**1**	**1**	-	**1**	-

**Total**	**1**	**5**	**34**	**22**	**2**	**17**	**17**	**18**	**9**	**17**

Note: One case of meningothelial meningioma did not have T1-W1 imaging performed, and the DWI-ADC and GRE sequencies were only performed in 85% (*n* = 35) and 63% (*n* = 26) of the cases, respectively.

Meningiomas showed associated parenchymal vasogenic oedema in the majority of cases (78.1%), which was most commonly found to be only mild to moderate (53.7%) (total *n* = 41). No statistically significant correlation between the Grade, perilesional oedema and specific histological subtype was identified (*p* = 0.37). One WHO Grade III meningioma showed severe perilesional oedema, but this was also encountered with 20% of WHO Grade I meningiomas (*n* = 7). The two WHO Grade II meningiomas showed mild to moderate perilesional oedema, which was also present with 55.8% of WHO Grade I meningiomas (*n* = 19). A summary of the data is presented in Table 4.

**TABLE 4:** Perilesional changes encountered with the varying histological subtypes of intracranial meningiomas.

Histological subtype	Perilesional findings associated with intracanal meningiomas							
	Perilesional brain oedema			Adjacent focal osseous changes			Dural Tail	
	Absent	Mild/moderate	Severe	Absent	Hyperostosis	Invasion	Absent	Present
**WHO grade I**								
Meningothelial	5	14	5	9	12	3	13	9
Transitional	2	3	-	2	2	1	1	4
Fibroblastic	-	1	-	-	1	-	1	-
Secretory	-	-	1	1	-	-	-	1
Psammomatous	-	1	-	1	-	-	-	1
Microcystic and angiomatous	-	-	1	1	-	-	1	-
Microcystic	1	-	-	1	-	-	-	1
Unspecified	1	1	2	2	2	-	3	1
**WHO grade II**								
Chordoid	-	1	-	-	-	1	-	1
Atypical	-	1	-	-	1	-	-	1
**WHO grade III**								
Anaplastic	-	-	**1**	**1**	-	-	**1**	-

**Total**	**9**	**22**	**10**	**18**	**18**	**5**	**20**	**19**

Note: Two cases of meningothelial meningioma did not have post-contrast imaging performed and could not be evaluated for a dural tail.

Almost half of the meningiomas (43.9%) showed normal adjacent bone without focal osseous changes, while hyperostosis was encountered in an equal number of cases (43.9%) and frank invasive or destructive changes in a minority of cases (12.2%) (total *n* = 41). Eighty per cent of the lesions associated with invasive bone changes were of the WHO Grade I histological subtype (*n* = 4); however, no statistically significant associations between types of osseous changes and different subtypes were identified (*p* = 0.58).

An identifiable dural tail was present in less than half of cases (48.7%) with postcontrast sequences (total *n* = 40). No statistically significant association between the presence or absence of a dural tail and a specific histological subgroup was identified (*p* = 0.31).

**FIGURE 2:**
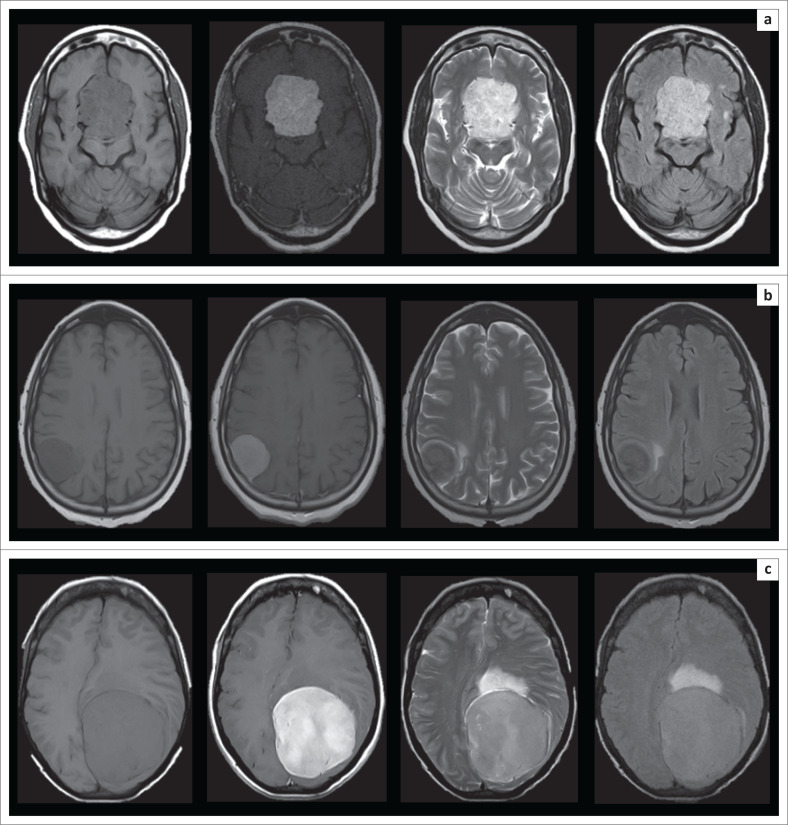
Selected axial MRI brain images depicting the appearances of the different common World Health Organization (WHO) Grade I meningioma subtypes encountered: (a) Meningothelial meningioma, (b) Transitional meningioma, and (c) Fibroblastic meningioma (shown from left to right on T1-weighted imaging [T1-WI], T1-WI post-gadolinium, T2-weighted imaging [T2-WI] and fluid attenuation inversion recovery [FLAIR] sequences).

**FIGURE 3:**
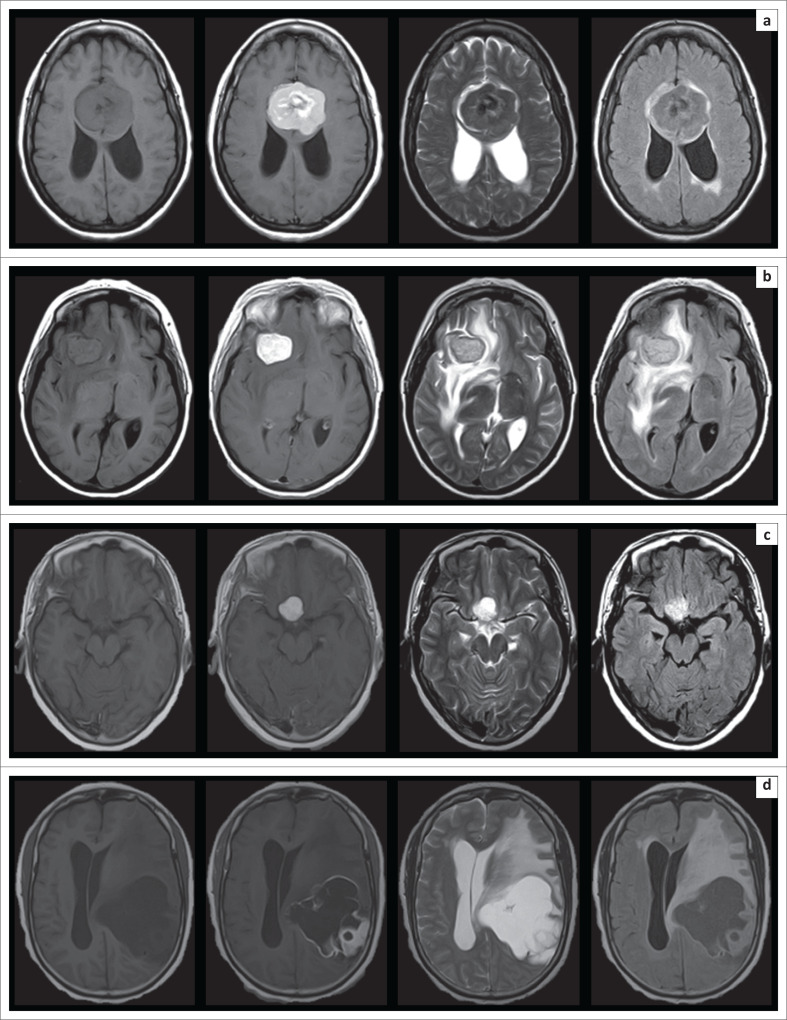
Selected axial MRI brain images depicting the appearances of the different World Health Organization (WHO) Grade I uncommon meningioma subtypes encountered: (a) Psammomatous meningioma, (b) Secretory meningioma, (c) Microcystic meningioma and (d) Mixed angiomatoid-microcystic meningioma (shown from left to right on T1-weighted imaging [T1-WI], T1-WI post-gadolinium, T2-weighted imaging [T2-WI] and fluid attenuation inversion recovery [FLAIR] sequences).

**FIGURE 4:**
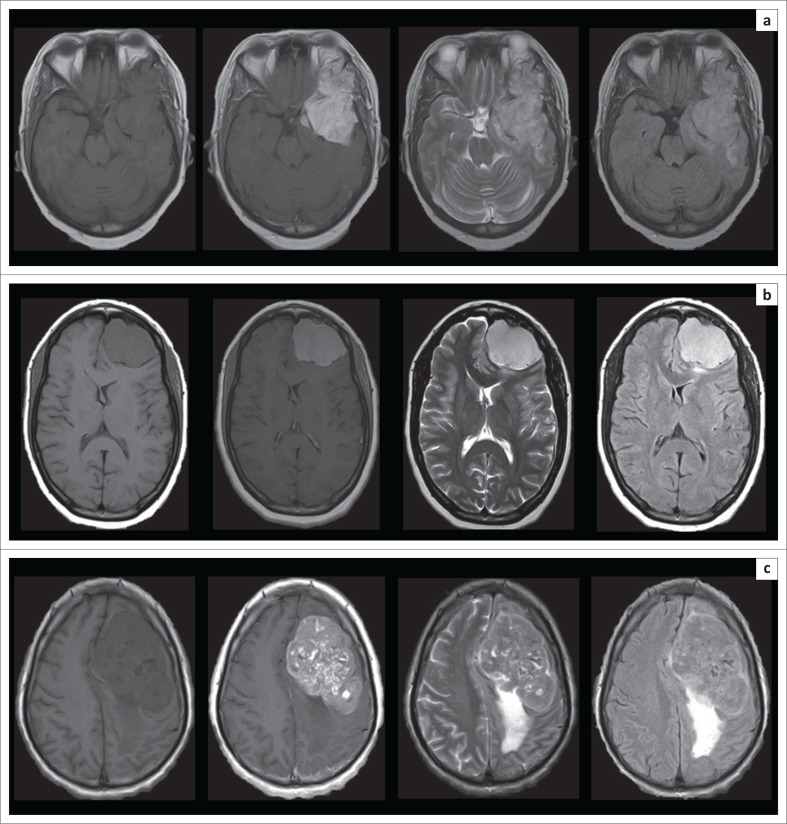
Selected axial MRI brain images depicting the appearances of the different World Health Organization (WHO) Grade II meningioma subtypes encountered: (a) Chordoid and (b) Atypical meningiomas, as well as the WHO Grade III subtype encountered, (c) Anaplastic meningioma (shown from left to right on T1-weighted imaging [T1-WI], T1-WI post-gadolinium, T2-weighted imaging [T2-WI] and fluid attenuation inversion recovery [FLAIR] sequences).

**FIGURE 5:**
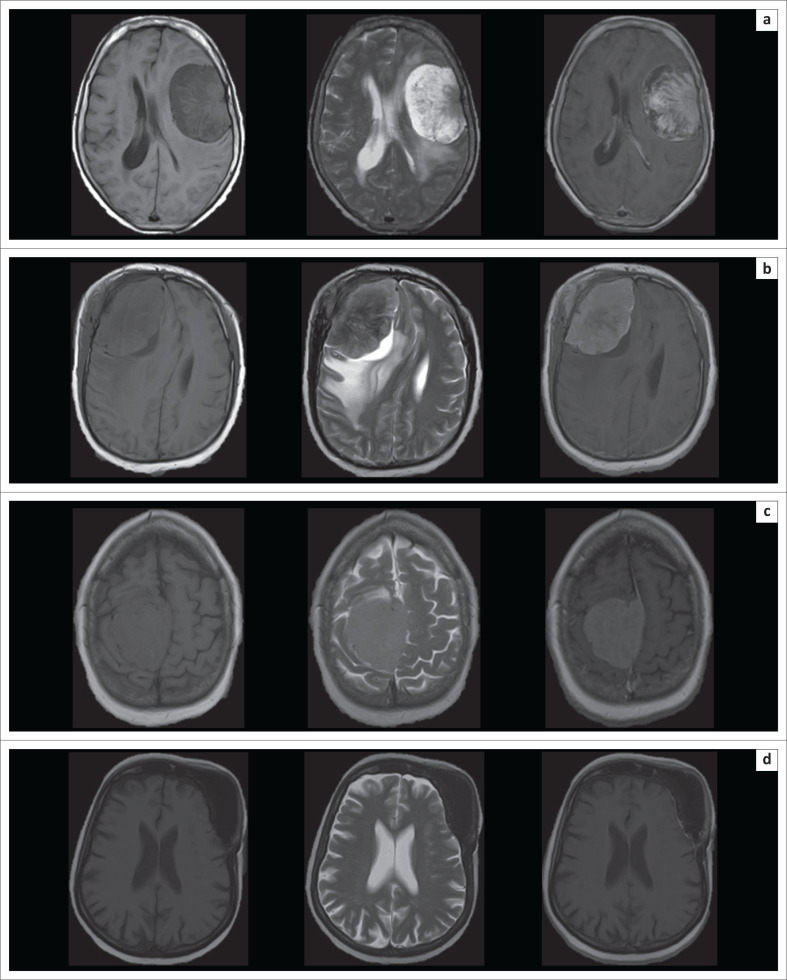
Selected axial magnetic resonance imaging brain imaging of various meningothelial meningiomas, highlighting the variability of features encountered among meningiomas sharing the same histological subtype. Image set a shows a T1-weighted imaging (T1-WI) hypointense, T2-weighted imaging (T2-WI) hyperintense mass lesion with somewhat heterogenous enhancement, and peripheral, intratumoural cyst formation. Image set b shows a predominantly T2-WI hypointense mass, with peritumoural cyst formation and moderate perilesional oedema. Image set c shows a mass lesion predominantly isointense on both T1-WI and T2-WI sequences. Image set d shows an en plaque morphology, with only a small, enhancing dural based lesion, associated with prominent underlying hyperostosis (shown from left to right on T1-WI, T2-WI and T1-WI post-gadolinium sequences).

The MR imaging appearances of the varying histological subtypes are presented in Figure 2, Figure 3, Figure 4 and Figure 5.

## Discussion

Meningiomas have a strong female predominance, which is also documented in this study, as well as in retrospective South African-based case reviews of 52 meningiomas by Fynn et al., 38 cases by Vivier et al. and 48 cases by Ibekuike et al.^5,6,7,8^ This is presumed to be because of the role of female sex hormones in modulating the growth of meningiomas; these lesions may express both progesterone and oestrogen receptors, and positive associations with pregnancy, hormone replacement therapy and breast cancer have been found.^9,10^

Meningiomas are classically considered to be tumours of the middle aged and elderly.^11,12^ A postulate for this is that because of more frequent imaging for concurrent neurological conditions in older patients, many clinically occult meningiomas are incidentally identified, which contributes to the overall higher incidence of these tumours as age increases.^10^ This trend was identified in this study; no cases were identified in patients younger than 30 years. The isolated cases of WHO Grade II and III meningiomas were also identified in patients 45 years and older. It bears mentioning that a recent South African study of 78 cases of meningioma from Motebejane et al. observed an increase in the prevalence of high-Grade meningiomas in patients 35 years and younger with HIV-1 co-infection.^12^ Although this result was not reproduced in the current study, it does, however, highlight the fact that previously well-established age-related trends may be changing in the context of the patient profile.

Large series analyses, including reviews of 1107 meningiomas by Sun et al. and 187 cases by Kamenova et al., identified the cerebral convexity as the most common intracranial location for meningiomas,^11,13^ in keeping with the findings of this study. Some studies, such as a large review of 729 cases of meningiomas by Bhat et al. found that WHO Grade II and III subtypes were more commonly identified at the skull base and intraventricular locations,^14^ which was not reproduced in the much smaller current study, where the anaplastic and atypical subtypes were found at the cerebral convexity. Meningiomas originate from arachnoid cap cells, which are more abundantly found in proximity to the venous sinuses as well as in skull base locations, a likely explanation for the predilection of meningiomas for certain sites.^15^

In general terms of histological assessment, the 2016 update of the WHO grading system for central nervous system (CNS) tumours retains the prior subdivision of meningiomas into three categories based on their histopathological features, which is further subdivided into 15 specific histological subtypes. The WHO Grade I meningiomas are most common by a substantial degree, reported in up to 88% – 94% of cases and comprise nine different histological subtypes, namely, meningothelial (syncytial), transitional (mixed), fibrous (fibroblastic) (the three common subtypes) lymphoplasmacyte-rich, microcystic, secretory, psammomatous, angiomatous and metaplastic meningiomas (these subtypes being uncommon). The WHO Grade II meningiomas consist of three histological subtypes, which are chordoid, clear cell and atypical meningiomas (much less frequently identified in only 5% – 7% of cases). The WHO Grade III meningiomas are the papillary, rhabdoid and anaplastic histological subtypes and are overtly malignant lesions, rarely seen in only 1% – 2% of cases.^1,16,17,18,19^ The internationally well-known predominance of WHO Grade I meningiomas was reproduced in the current study and is comparable to many others, including a recent large single institutional study of 163 meningiomas from Africa, based in Nigeria, by Salami et al.^7^ Their study, however, identified translational meningiomas as the most common in their population, which is comparable to a recent study of 224 meningiomas by Ihwan et al., which also found translational meningiomas as the most common histological subtype in their population.^7,20^ These findings are in contrast to the current study, which identified the meningothelial subtype as the most common by far, in keeping with international literature, such as a large analysis of 729 cases by Bhat et al.^14^

In terms of overall morphology, the classic imaging description of a globose, well circumscribed and avidly enhancing extra-axial mass lesion was applicable to the majority of the cases of this study, irrespective of the subtype.^1,17^ In the literature, en plaque meningiomas are far less common, reported to comprise between 2% and 9% of cases, usually identified in the spheno-orbital region, and commonly associated with invasion of underlying bone with concurrent, prominent hyperostosis. Of interest is that, despite their poorly understood inherent locally invasive nature, en plaque meningiomas are usually WHO Grade I meningiomas because of their low index of cellular proliferation and commonly of the meningothelial subtype.^21,22^ These features were reproduced in this study, with a low incidence of en plaque meningiomas, poorly circumscribed margins at their interface with the underlying bone (75%) and a strong association with hyperostosis (100%); most were located at the sphenoid wing (75%) and all were of the meningothelial histological subtype.

As expected, most meningiomas were homogenous. The heterogeneity encountered in a small proportion of lesions was predominantly because of calcification and cystic components. Calcification is an inconsistent feature of meningiomas, best demonstrated on CT.^1,23^ Cystic components associated with meningiomas may be intrinsic because of degeneration or entrapped cerebrospinal fluid, or extrinsic, related to reactive arachnoid cyst formation.^3^

Although meningiomas are vascular lesions in general, this may not be reflected by the presence of discernible flow voids on conventional MRI sequences and is more optimally assessed on conventional angiography or MR perfusion studies. The overall paucity of internal vascular flow voids is in keeping with the predominance of common benign Grade I meningiomas; higher Grade meningiomas are expected to have a higher degree of tumoural angioneogenesis.^3,24^

Mild to moderate perilesional vasogenic oedema was a common finding in the majority of meningioma cases in this study, especially with common WHO Grade I subtypes, in keeping with literature.^25^ This highlights the fact that the presence of peritumoural oedema itself is a poor overall predictor of tumoural vascularity or histological subtype.^3^ In this study, the severity of perilesional oedema was also not a strong predictor of uncommon or malignant subtypes, with 63% of cases with severe oedema (*n* = 5) seen with the common meningothelial subtype. The dural tail sign, a hypervascular, reactive and non-neoplastic dural reaction, is not pathognomonic for meningioma,^26,27^ and the correlation between the presence of a dural tail and specific histological subtypes lacks extensive research. The presence of a dural tail has been reported in up to 72% of cases of WHO Grade I meningiomas.^15^ In terms of osseous changes, bone invasion is an uncommon finding but is encountered with all WHO grades and may serve as a potential predictor of local recurrence. A large study of 1469 cases by Lemée et al. showed bone invasion in 18.7% of meningiomas, comparable to the current study with 12.2% of cases.^28,29^ Reactive hyperostosis is a more common finding unrelated to the size of the tumour. It is variably reported in up to 20% of cases in some studies,^3^ and usually most exuberantly seen with en plaque morphologies. The proposed mechanism is tumoural associated periosteal hypervascularity, which leads to the formation of new bone. In this study, a degree of hyperostosis was seen mostly with meningothelial meningiomas, irrespective of the morphology. However, it is worth observing that bone changes may be more optimally assessed on CT.^1^

Classically, meningiomas have been described as isointense to the cerebral cortex on all conventional MRI sequences.^1,15^ The T1-WI and T2-WI signal intensity characteristics of the meningiomas in this study were comparable with the findings of an analysis of 35 cases of meningioma by Mairuri et al.^4^ Their study found that the majority of lesions (71.5%) were isointense to grey matter on T1-WI, irrespective of histological subtype, and that meningiomas commonly showed both T2-WI isointensity (43%) and hyperintensity (40%) in relatively equal numbers of cases.^4^ However, the current study showed no statistically significant correlation between the T2-WI signal of the lesions and their histological subtype, with the most common meningothelial subtype showing T2-WI hyperintensity in a large number of cases.

In terms of specific appearances of distinct subtypes, a variety of isolated cases of uncommon histological subtypes were encountered (examples of all encountered subtypes are displayed in Figure 2, Figure 3 and Figure 4). An isolated psammomatous subtype was present. These lesions are characterised by extensive calcification, which characteristically produces low T2-WI signal, yet they commonly maintain postcontrast enhancement, as in the present case.^30^ An isolated fibroblastic subtype was present, which demonstrated a solid, homogenous and non-vascular appearance, in keeping with the literature; however, the T2-WI signal of these lesions has been variably reported in the literature as unlikely to be T2 hyperintense^4^ reflecting not only their hard, fibrous consistency but also as commonly T2 hyperintense because of their spindle cell proliferation, as in the present case.^31^ Both pure microcystic and mixed microcystic-angiomatoid subtypes were present. Microcystic meningiomas have been reported as the rarest of all subtypes and were only recognised by the WHO as distinct entities in 1993.^32^ These uncommon, benign WHO Grade I meningiomas are characterised by high T2-WI signal intensity, as well as severe peritumoural oedema, mimicking more aggressive neoplasms.^33^ Similar features are shared by secretory meningioma subtypes, one of which was encountered. These lesions are also known for their severe degree of perilesional oedema,^34^ which was present in the single case identified in this study. The degree of oedema can commonly increase rapidly in the post-operative setting and can lead to life-threatening intracranial herniation syndromes.^35^ An isolated chordoid subtype was present; these seldom encountered WHO Grade II variants comprise only about 0.5% of total meningioma cases, are known to mimic chondroid lesions on imaging, and can exhibit aggressive behaviour with an increased likelihood of recurrence post resection.^36^ A case series analysis of these tumours by Pond et al. in 2015 described them as most consistently appearing T2 hyperintense and T1 isointense, with avid postcontrast enhancement, as in the present case.^37^ They most characteristically show facilitated diffusion with increased signal on the ADC map^37^; this study case did not show significant diffusion facilitation or restriction. An isolated WHO Grade II atypical variant was encountered; these lesions classically show heterogenous T1-WI and T2-WI signal intensity, prominent enhancement, significant perilesional oedema and a poor plane of separation from adjacent brain tissue;^38^ however, this study case did not demonstrate any of these suspicious features, but instead showed a circumscribed, homogenous, solid and well marginated lesion with only mild perilesional oedema. An isolated case of anaplastic meningioma was also present. A fact sometimes overlooked and under-investigated is that these rare meningiomas can metastasize to extracranial locations, reported in up to 16.6% of cases in a recent retrospective review by Seo et al.^39^ and carry an overall poor prognosis. These tumours may exhibit suspicious features; however, some studies showed no significant differences in appearance of benign and anaplastic subtypes.^4^ Enhancement of these lesions has been described as moderate and heterogenous rather than an expected avid pattern.^31^ This study’s case of anaplastic meningioma reflected these relatively indolent characteristics, with heterogeneity because of presumed calcification, supported by significant blooming on GRE, and otherwise isointense components seen across T1-WI, T2-WI and FLAIR sequences, as well as a mild to moderate septal pattern of postcontrast enhancement. However, severe perilesional oedema was present. This study did not identify lymphocytoplasmic-rich, metaplastic, clear cell, papillary or rhabdoid meningioma subtypes.

### Study limitations and recommendations

Inherent bias is present in the study population, as patients with small or asymptomatic meningiomas do not routinely present for imaging workup. Asymptomatic or small meningiomas, lesions in anatomic locations with difficult operative accessibility and patients with high anaesthetic risk would likely not have been candidates for resection or biopsy and could thus not be included. These factors could account for the relatively small study population, which limits the statistical significance of the results. The study was also a single-centre study, performed at a smaller tertiary centre. Additional factors limiting the number of patients undergoing operative intervention may be challenging socioeconomic circumstances leading to impaired patient follow-up, as well as the study period partially coinciding with the COVID-19 pandemic, limiting access to both elective MRI and surgery.

Standardising and implementation of more advanced imaging protocols at tertiary institutions is also a consideration; however, the additional benefit in terms of specific diagnostic accuracy should be weighed against the cost of prolonged imaging times in the resource-limited South African setting. Such protocols could include routine magnetic resonance spectroscopy (MRS), where Grade I meningiomas usually exhibit high alanine and choline peaks, high alanine:creatine (Cr) ratios and decreased N-acetylaspartate (NAA), features that have been shown to aid in diagnosis, but again has not been reliably found to allow distinction between the different histological subtypes.^15^ A recent study by Panirahi et al. showed promise specifically by using ADC values, MR spectroscopy and MR perfusion imaging with the aim of differentiating WHO grades and with subtyping of typical meningiomas.^40^

## Conclusion

The T1-WI and T2-WI signal intensity of meningiomas in this study’s population are comparable to findings from other studies of similar sizes. An analysis of this case series of intracranial meningiomas did not demonstrate statistically significant differences in MRI features useful to differentiate between histological subtypes of meningioma, or to allow for accurate preoperative prediction of WHO grade. This highlights the importance of definitive histopathological diagnosis rather than overreliance on presumed benign imaging features. A variety of uncommon histological variants were encountered with unique imaging characteristics, necessitating familiarity with these lesions to enable accurate diagnosis. The most commonly identified subtype of meningothelial meningioma is in keeping with international literature but differs from what was found in a large African study, highlighting the importance of further population specific research.
